# Draft Genome Sequence of a Wohlfahrtiimonas chitiniclastica Strain Isolated from Frozen Chicken in Rio De Janeiro, Brazil

**DOI:** 10.1128/MRA.00352-19

**Published:** 2019-12-05

**Authors:** Jorge Matos, Adriana Rocha Faria, Ana Paula D’Alincourt Carvalho Assef, Ângela Corrêa de Freitas-Almeida, Rodolpho Mattos Albano, Mara Lucia Penna Queiroz

**Affiliations:** a Departamento de Microbiologia, Imunologia e Parasitologia, Faculdade de Ciências Médicas (FCM), Universidade do Estado do Rio de Janeiro, Rio de Janeiro, RJ, Brazil; b Departamento de Bioquímica, Instituto de Biologia Roberto Alcântara Gomes (IBRAG), Universidade do Estado do Rio de Janeiro, Rio de Janeiro, RJ, Brazil; c Faculdade de Ciências Médicas, Universidade Federal do Rio de Janeiro, Rio de Janeiro, RJ, Brazil; d Laboratório de Pesquisa em Infeccão Hospitalar (LAPIH), Instituto Oswaldo Cruz, Rio de Janeiro, RJ, Brazil; University of Maryland School of Medicine

## Abstract

Here, we report the draft genome sequence of Wohlfahrtiimonas chitiniclastica strain 20, isolated from a chicken carcass originated from indoor broiler farming and identified using matrix-assisted laser desorption ionization–time of flight (MALDI-TOF) mass spectrometry followed by sequencing of the 16S rRNA gene.

## ANNOUNCEMENT

Wohlfahrtiimonas chitiniclastica is a Gram-negative bacillus that lives in the larvae of the parasitic flies Wohlfahrtia magnifica and Chrysomya megacephala (1, 2), and it has been reported as the causative agent of severe infections in clinics and as a threat to the health of humans and animals. This bacterium was found to be a potential zoonotic pathogen associated with human bacteremia and sepsis (3–7), with dolphin endocarditis (8), with white-tailed deer septicemia (9), and with cattle hoof infection (10).

In this study, we describe the draft genome sequence of W. chitiniclastica strain 20, isolated for the first time from a chicken carcass as previously described (11). A single bacterial colony grown on tryptone soya agar (Oxoid, United Kingdom) was transferred to tryptone soya broth (Oxoid) and incubated overnight at 37°C. After that, genomic DNA was extracted using a NucleoSpin tissue kit (Macherey-Nagel, Germany). Genomic libraries were constructed with the Nextera XT DNA sample prep kit (Illumina, Inc.). Sequencing was performed on an Illumina MiSeq system with the 600-cycle MiSeq v3 reagent. Paired-end reads (1,681,842) were trimmed, corrected, and assembled with A5-miseq pipeline (v. 20140604) using default parameters (12) to produce 11 scaffolds (12 contigs) comprising a genome of 2,180,519 bp with 43.67% GC content and an 11,348-bp plasmid. The longest scaffold was 1,074,268 bp, the *N*_50_ value was 427,544 bp, the *L*_50_ value was 2, the raw coverage was 299×, and the medium coverage for both the chromosome and the plasmid was 430×. The plasmid was detected by assembling the reads with plasmidSPAdes with default parameters (v. 3.10.1) (13).

Genome and plasmid annotations were performed with the NCBI Prokaryotic Genome Annotation Pipeline (14) and the RAST server v. 2.0 (15). The presence of plasmid stabilization, replication, and conjugation proteins helped to confirm the identity of the plasmid. Resources available at the Pathosystems Resource Integration Center (PATRIC) v. 3.4.11 were also used in the default mode for annotation (16). Preliminary draft annotation indicates that the genome is predicted to contain 2,080 genes encoding 1,987 proteins, 50 tRNAs, and 9 rRNAs. The genome also contains genes coding for macrolide-specific efflux pumps (*macA* and *macB*). We also observed the presence of the *oxyR* gene, which is involved in regulating oxidative stress resistance. The presence of one 25.9-kb intact phage in the genome was shown by a search with the PHAST engine (default mode; http://phast.wishartlab.com/), which revealed the highest similarity with *Enterobacteria* phage Fels-2 (GenBank accession number NC_010463), which was initially described in Salmonella enterica LT2 (17). Two sequences associated with CRISPR regions were identified using CRISPRfinder in the default mode (https://crispr.i2bc.paris-saclay.fr/Server/) (18), presenting a total of 246 CRISPR repeats and 244 CRISPR spacers. The reciprocal average nucleotide identity (ANI) index (19) between strain 20 and three other strains of *W. chitiniclastica*, SH04, DSM18708, and BM-Y, reveals that strain 20 shares 96.9%, 98.1%, and 98% identity with SH04, DSM18708, and BM-Y, respectively.

### Data availability.

The whole-genome shotgun for this project has been deposited at DDBJ/ENA/GenBank under the accession number NZ_LWST00000000. The version described in this paper is version NZ_LWST00000000.1. Raw sequence reads have been deposited in the NCBI Sequence Read Archive (SRA) with accession number SRX1733470 under BioProject number PRJNA224116.
